# *In silico* analysis of the *Mus musculus* uterine gene expression landscape during pregnancy identifies putative upstream regulators for labour

**DOI:** 10.1371/journal.pone.0204236

**Published:** 2018-09-20

**Authors:** Febilla Fernando, Souad Boussata, Aldo Jongejan, Joris A. van der Post, Gijs Afink, Carrie Ris-Stalpers

**Affiliations:** 1 Reproductive Biology Laboratory Amsterdam UMC, University of Amsterdam, Amsterdam, The Netherlands; 2 Department of Bioinformatics, Amsterdam UMC, University of Amsterdam, Amsterdam, The Netherlands; 3 Women’s and Children’s Clinic, Department of Obstetrics and Gynaecology, Amsterdam UMC, University of Amsterdam, Amsterdam, The Netherlands; University of Missouri Columbia, UNITED STATES

## Abstract

**Background:**

The molecular pathways involved in the transition from uterine quiescence to overt labour are mapped and form the currently established pharmacological targets for both the induction and inhibition of human labour. However, both spontaneous premature labour and functional dystocia occur and are difficult to treat adequately. The identification of upstream regulators involved in the onset and orchestration of labour pathways is essential to develop additional therapies that will contribute to the regulation of the timing of birth.

**Objectives:**

To define uterine biological processes and their upstream activators involved in the transition from uterine quiescence to overt labour.

**Study design:**

The uterus of non-pregnant and pregnant FVB *M*. *musculus* is collected at embryonic days (E) 6.5, 8.5, 10.5, 12.5, 15.5 and 17.5 and the uterine transcriptome is determined using the Illumina mouse Ref8v2 micro-array platform. K-means clustering and Ingenuity Pathway Analysis are applied to further dissect the transcriptome data.

**Results:**

From E6.5 to E17.5, 5405 genes are significantly differentially expressed and they segregate into 7 unique clusters. Five of the 7 clusters are enriched for genes involved in specific biological processes that include regulation of gene-expression, T-cell receptor activation, Toll-like receptor signalling and steroid metabolism. The identification of upstream activators for differentially expressed genes between consecutive time points highlights the E10.5 to E12.5 window during which the role from progesterone switches from an activated state to the inhibited state reflecting the process of functional progesterone withdrawal essential for the transgression from myometrial quiescence to synchronized contractions. For this time window in which 189 genes are differentially expressed we define 22 putative upstream activators of which NUPR1 and TBX2 are the most significant with respectively an activated and an inhibited status.

**Conclusions:**

Gene expression profiling of mice uterus from E6.5 to E17.5 results in 7 unique gene expression clusters from early to late pregnancy that define the landscape of molecular events in ongoing pregnancy. In the current dataset progesterone is predicted as an activated upstream regulator and maintainer of myometrial quiescence and is active till E10.5. Progesterone is predicted as an inhibited upstream regulator at E12.5. We identify 22 upstream regulators in the E10.5 to E12.5 time window where the switch to progesterone withdrawal occurs. They are putative relevant upstream activators of labour.

## Introduction

The transition from uterine quiescence to overt labour in placental mammals is marked by functional progesterone withdrawal, induction of prostaglandins, increase of pro-inflammatory cytokines and interleukins, activation of the oxytocin receptor by oxytocin and increase of voltage gated Ca^2+^ channels facilitating contractions [1]. These processes have been investigated in detail and are rational and established therapeutical targets to intervene in the process of human labour when it either occurs preterm and/or does not advance properly as in functional labour dystocia.

Functional dystocia is defined as inadequate uterine activity where delivery fails to progress during the active phase of labour [2, 3]. Functional dystocia highly contributes to caesarean rates in nulliparous women [4, 5]. early interventions such as the administration of oxytocin only result in a clinically modest reduction in the caesarean section rate [6]. This implies that further activation of the oxytocin receptor only is not sufficient to generate strong synchronized contractions during the active phase of labour.

Preterm birth, defined as delivery before the 37th week of gestation, is the most common cause of neonatal mortality and the second leading cause of death in children under five years of age [7]. Preterm birth is associated with immediate and long term morbidity as well as growth and developmental delay [8, 9]. Progesterone treatment, counteracting the functional progesterone withdrawal, or administration of cyclooxygenase inhibitors to inhibit prostaglandin synthesis, do not substantially reduce the risk of preterm birth [10–13]. Although oxytocin receptor antagonists and calcium channel blockers are effective in delaying preterm delivery (~7 days) [14–16] the majority of the women (~65%) receiving this treatment still deliver preterm [17]. Research on understanding mechanisms governing parturition initiation has recently escalated owing to the increasing rates of preterm birth around the globe over the last 2 decades [18].

In the current study we aim to identify upstream regulators with a putative role in triggering molecular pathways relevant for the synchronized contractions necessary to attain a timely vaginal delivery.

In humans, it is unethical and thus impossible to track the uterine gene expression changes throughout gestation in relation to the initiation of parturition, making it necessary to use animal models. All animal models used to study human parturition have their limitations [19]. Because of their relative short gestation varying from 18.5 to 20.5 days, their fully available genome sequence and the opportunity to harvest uterine tissue during gestation, mice are an ideal species to perform gestational gene expression studies. It has been demonstrated that mice can mimic human pathologies with respect to the infection induced preterm birth [20–23].

Functional withdrawal of progesterone is essential for the progression from quiescence to overt labour. While maternal progesterone levels in rodents decline near term, in humans they remain high till birth and is it the switch of the highly bioactive B isoform of the progesterone receptor to a less bioactive A isoform that contributes to functional progesterone withdrawal [24]. This classic distinction between man and mice is less absolute then sometimes reported. The declined maternal progesterone levels in mice towards term remain well above the dissociation constant for binding to the progesterone receptor and the switch in progesterone receptor isoform expression also occurs in mice [25, 26]. In addition, functional progesterone withdrawal is in part achieved by increased local progesterone metabolism, a process similar in mice and human [26].

The downstream effects of functional progesterone withdrawal are induction of prostaglandins, pro-inflammatory mediators, expression of gap junction protein alpha 1 (connexin-43) and the oxytocin receptor. Although these processes in mice parturition are also not fully identical to human parturition there are demonstrable similarities [1, 19, 25–29]. Uterine specific knockout of *Trp53* in mice results in both preterm birth and dystocia [30]. Previous RNA microarray studies on mouse uterus have mostly focused on selective time points during late gestation establishing pathways relevant to labour that are similar to those also relevant for human parturition [23, 26, 31–35].

Gene expression related time course studies on a prolonged earlier time periods of mouse gestation have not yet been reported and they are essential to define novel upstream triggers for labour associated pathways. The utilization of varying gestational time points, mice strains, experimental setups, interventions and array platforms used makes the merging of existing gene expression data extremely difficult and hampers the identification of potential upstream regulators.

In the current study, we define the gene expression patterns in FVB mouse uterus across gestation from E6.5 to E17.5. We identify gene expression clusters and define novel putative upstream regulators for labour.

## Materials & methods

### Tissue collection and sample preparation

All experimental procedures in this study were approved by the Animal Ethics Committee of the Academic Medical Center, Amsterdam, The Netherlands (Permit Number: DVF102563). All methods were performed in accordance with relevant guidelines and regulations. FVB/N mice were purchased from Harlan Laboratories. Staged embryos were obtained by crossing FVB/N mice and checking the next morning for a vaginal plug. The morning of discovery of the vaginal plug was designated as embryonic day (E) 0.5.

Mice samples were collected after termination by carbon dioxide inhalation from non-pregnant (NP)and pregnant FVB mice at E6.5, E8.5, E10.5, E12.5, E15.5 and E17.5 respectively. NP mice were taken at random. Median ages were 81 days (range 56–146) for non-pregnant and 79 days (range 61–99) for pregnant mice. Uterine tissue from pregnant mice was collected at implantation sites. Embryonic/placental tissue was removed and decidua was scraped of using a sterile blade. Uterine tissue was immediately placed into RNAlater and processed according to the instruction of the manufacturer. Samples were stored at -80°C until further use. Total RNA from uterine tissue samples was isolated using Trizol reagent (Thermo Fischer Scientific; 15596026) after lysing the tissues using the MagNA lyser (Roche). Isolated total RNA was cleaned up using RNA easy mini kit (Qiagen; 74104). The integrity of the RNA was checked using the Bioanalyser (Agilent). Because of a poor RIN score (RIN <7), one tissue sample from E6.5 and one tissue sample from E8.5 were excluded for further downstream use. For each individual mouse, total RNA samples with a RIN score greater than 7 were pooled. In total 32 total RNA samples, NP (n = 4), E6.5 (n = 4), E8.5 (n = 4), E10.5 (n = 5), E12.5 (n = 5) E15.5 (n = 5) and E17.5 (n = 5) were labelled using the Illumina total prep RNA amplification kit (AMIL1791) with an input of 200ng/μl.

### Microarray hybridization

Illumina Mouse Ref8 platform was used to establish gene expression patterns of the current dataset. The hybridization of the microarray was performed by Service XS (Leiden, The Netherlands). For array hybridization 1 ug of total RNA of the uterus samples was used and samples were randomized over arrays.

### Microarray data preprocessing and analysis

The data analysis was performed using the statistical software package R (version 3.3.2). The quality of the dataset was checked using arrayQualityMetrics (version 3.30.0) for 3 main parameters: comparison between arrays, array intensity distributions, and individual array quality. No outlying samples were identified. Probes (5528) with a non-significant detection P value of >0.05 on all 32 arrays were filtered out and not used for subsequent analysis. Normalization was performed on 32 samples starting from the Illumina sample and control probe profiles using the normexp-by-control background correction, quantile normalization, and log2 transformation from the limma package (v3.32.2). If multiple probes were mapped to the same Entrez Gene identifier according to the illuminaMousev2.db package (v1.26), the probe with the highest standard deviation was chosen. Separate contrasts were constructed for calculating differential expression between consecutive time points (NP vs E6.5, E6.5 vs E8.5, E8.5 vs E10.5, E10.5 vs E12.5, E12.5 vs E15.5 and E15.5 vs E17.5). Differential expression between the experimental conditions (time points) was assessed with a moderated t test using the linear model framework from the limma package. Resulting P values were corrected for multiple testing using the Benjamini-Hochberg false discovery rate. Corrected P values ≤0.05 were considered statistically significant. The resulting significant genes were used for prediction of upstream regulators. The gap statistic method (clusGap R function-cluster package v.2.0.5) was used to determine the optimal n number of clusters based on the firstSEmax value. Subsequently, K-means clustering (default algorithm) was used to obtain gene clusters using the scaled normalized relative gene expression data on a log2 scale for each expressed gene using packages clValid (v0.6–6) and cluster (v2.0.2).

### Gene ontology and pathway enrichment analysis

The gene ontology (GO) Biological Process (2017) enrichment function provided by the Enrichr program [36, 37] was used for GO enrichment of all genes in each cluster. The GO enrichment was considered significant if it had a Benjamini-Hochberg adjusted P value less than 0.05. Pathway enrichment analysis for consecutive time point comparisons was done using the Enrichr program [36, 37] web based tool and the WikiPathways 2016 module.

### Prediction of upstream regulators

Ingenuity pathway analysis (IPA) [38], an approach considering experimental observations in any species, tissue or cell type, was utilized to predict upstream regulators of the DEGs between consecutive embryonic time points. The significant (differential genes across consecutive time points (DEGct) from every comparison (FDR<0.05) was imported into IPA as separate text files with gene symbols and log2 fold change values. Upstream regulators prediction was based on the direction of fold change of the genes using MouseRef8 as gene background. The upstream regulators were considered significant if they had a significant prediction Z score greater than 2. The program gives a P value of overlap with the downstream targets. The Z scores can independently predict the activation or inhibition of an upstream regulator independent of the p value of overlap.

### Data repository

The microarray data have been deposited in the NCBI Gene Expression. Accession number GSE85934.

## Results

### Quality control and normalization

**Fig 1:**
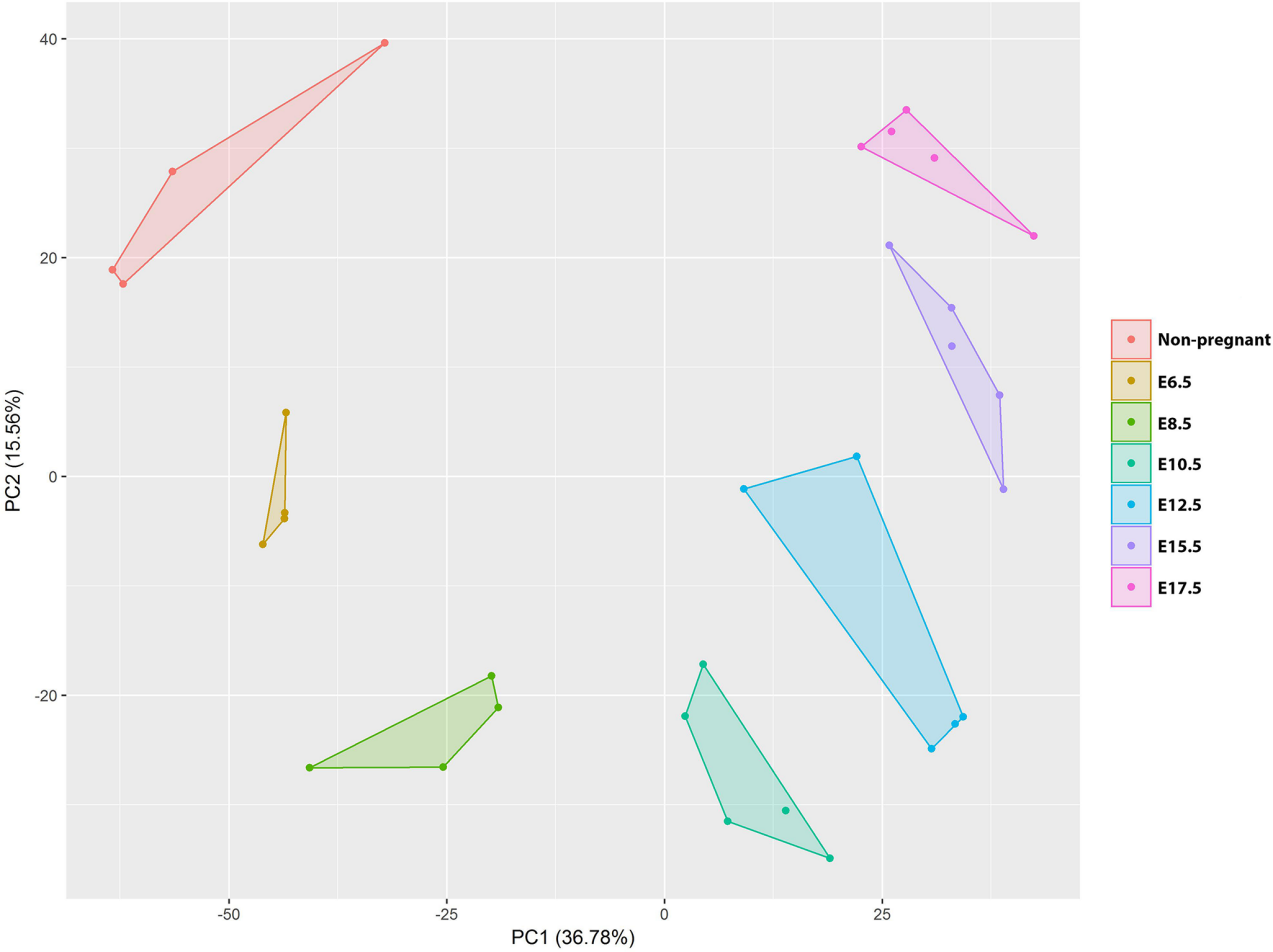
Principal component analysis (PCA). PCA of the quantile normalized dataset consisting of 32 mouse uterine RNA samples. The colours represent different embryonal days at which the tissue was harvested. % of total variance for PC1 and PC2 is indicated between brackets.

The expression profiles of in total 32 samples (with a minimum of 4 different mice per time point) were analysed on Illumina mouse Ref8v2 micro-array platform. Principal component analysis (PCA) shows that uterine tissue samples harvested during same embryonic time points cluster together, indicating significant gestational age based gene expression differences between time points (Fig 1).

### Gene expression clusters during gestation from E6.5 to E17.5

To define patterns of gene expression during gestation, differential gene expression was calculated between a minimum of two consecutive or non-consecutive time points across gestation (DEGots). Applying an adjusted p value filtering <0.05, 5405 DEGots are identified. With a 1.5 log fold change (lfc) cut-off this reduces to 971 DEGots and with a 2 lfc cut off that number further decreases to 520. To comprehend the entirety of gene expression networks and associated molecular pathways, the 5405 significant DEGots with no additional lfc were used for clustering and subsequent gene ontology enrichment analysis.

**Fig 2:**
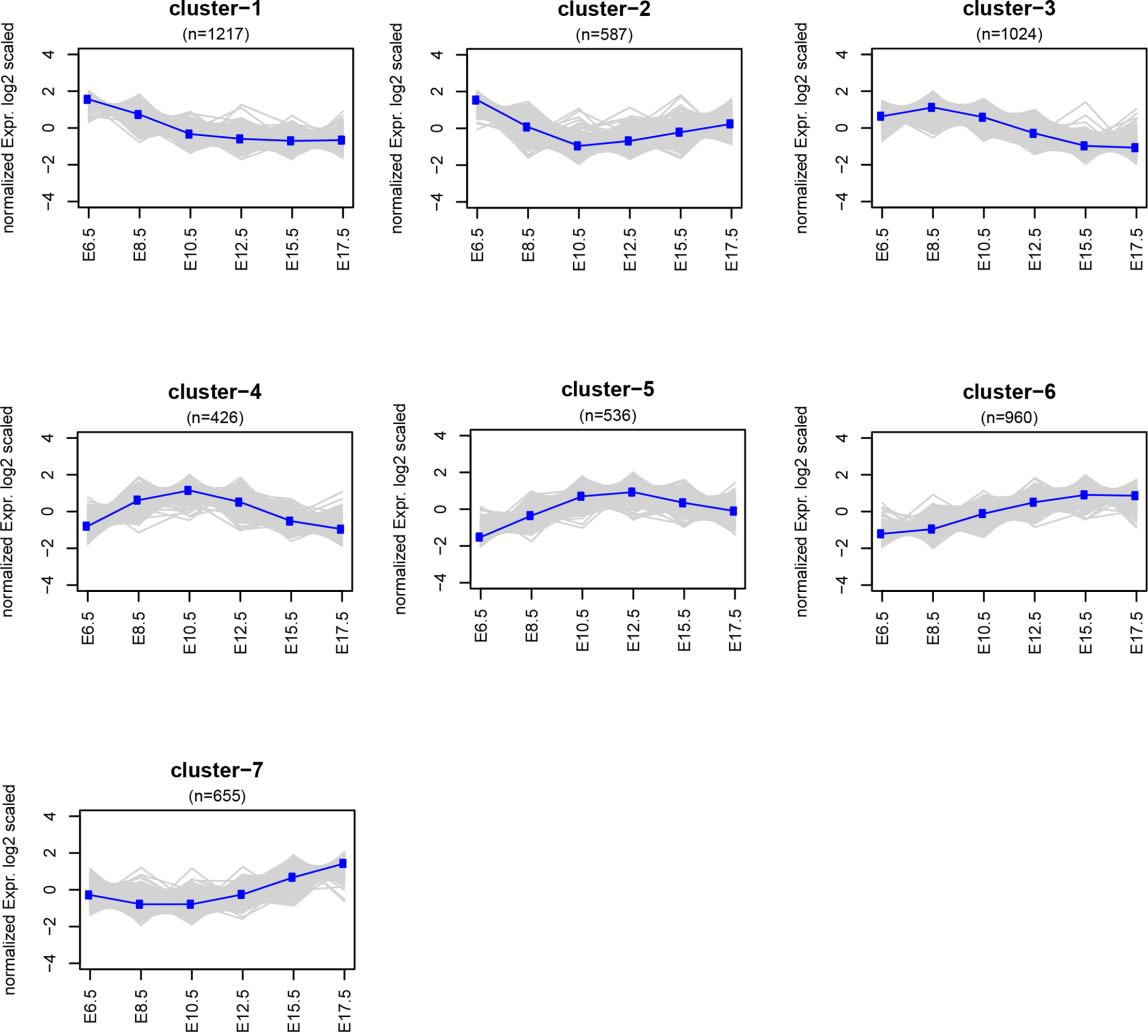
K-means cluster analysis. K-means clustering of differentially expressed genes changing over time (DEGots) from E6.5 to E17.5. The 5405 DEGots are clustered by K-means algorithm. n: number of genes in each cluster.

The gap statistic method [39] shows that the highest fraction of variance between differential gene expression patterns can be explained by 7 clusters (S1 Fig and Fig 2, S1 Table). Cluster 1 and cluster 2 show relatively high expression at E6.5. Cluster 2 shows a slight increase of expression towards later pregnancy, which differentiates it from cluster 1. Both clusters 1 and 2 do not display any significant (adjusted P-value < 0.05) enrichment of gene ontology (GO) terms. Cluster 3 gradually decreases from E8.5 to late pregnancy and is significantly enriched in processes involved in cytoplasmic translation and ribosome biogenesis. Cluster 4 and 5 have an arc shaped pattern with an increase from early-to mid-pregnancy and decrease from mid-to-late pregnancy. Cluster 4 is enriched in T cell receptor signalling and regulation of steroid biosynthesis with decreasing expression after E10.5. In cluster 5 expression levels drops after E12.5 and it is enriched in neutrophil activation. Cluster 6 increases from early to late pregnancy and is enriched in neutrophil activation, protein metabolism, Toll-like receptor signalling pathway/activation of immune response and apoptotic cell clearance. Cluster 7 shows an increase from mid to late pregnancy and is enriched in regulation of protein phosphorylation, protein-lipid complex remodelling and sterol transport (S2 Table). Clusters were evaluated with respect to the presence of genes well established as having a role in the transition from uterine quiescence to synchronized labour relevant for cortisol bioactivity (*Hsd11b1 and Hsd11b2*) [40], functional progesterone withdrawal (*Stat5b*, *Zeb1*, *Zeb2*,) [35, 41], the oxytocin receptor (*Oxtr*) and the *Gja1* gene encoding the gap junction protein connexin 43 [42]. Cluster 3 with decreasing expression from early to late pregnancy contains *Gja1*. Cluster 1, with relatively high expression during early pregnancy that decreases as pregnancy advances, contains the *Zeb1* and *Hsd17b1* genes. Cluster 6, with overall increasing expression levels from E6.5 to E17.5, contains *Hsd11b1* and the transcription factor *Elf3* that has previously been shown to increase transcription from the *Ptgs1* promoter [33].

### Identification of upstream regulators using DEGcts as downstream gene targets

**Fig 3:**
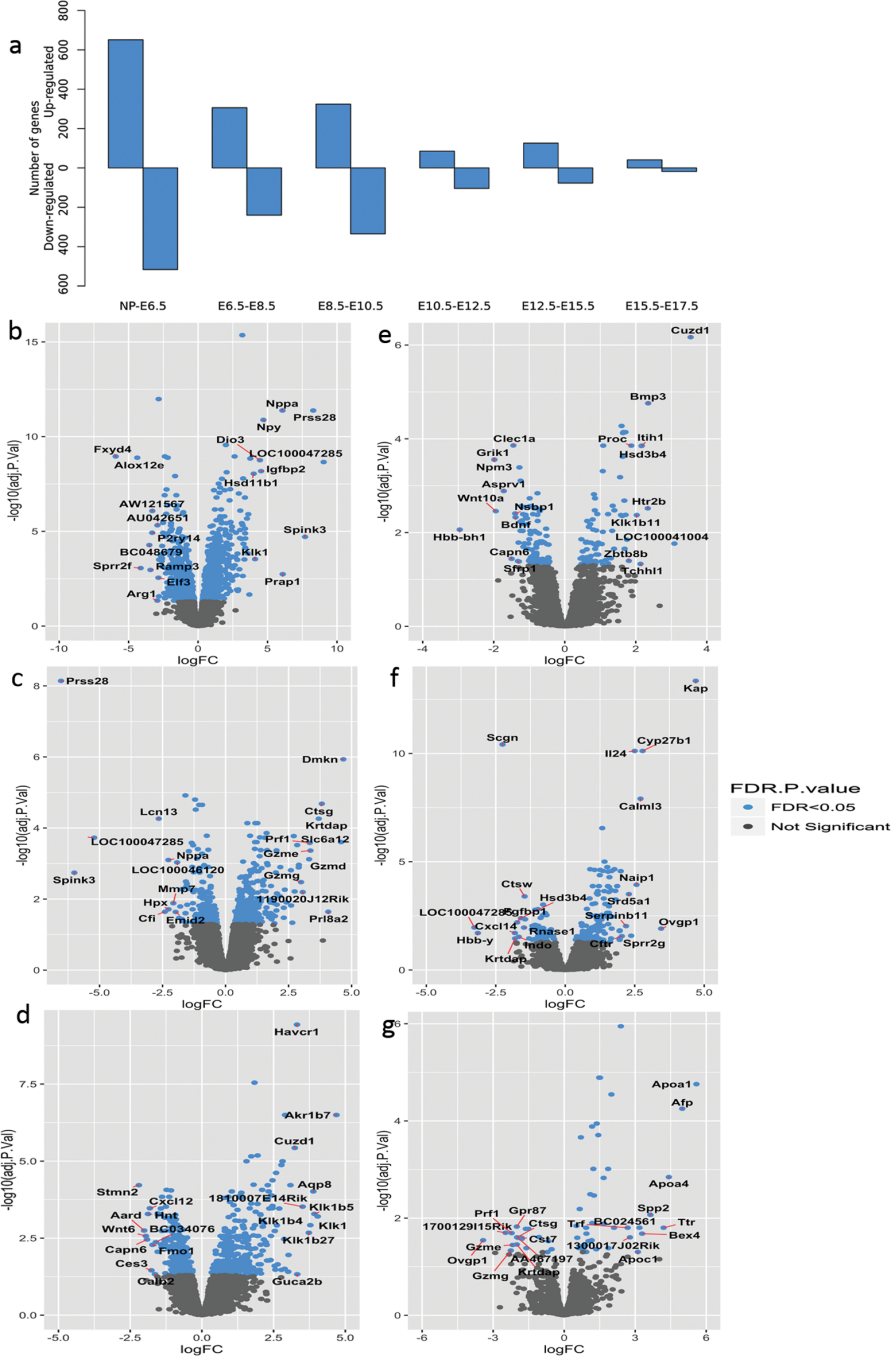
Differentially expressed genes between consecutive time points (DEGcts). a: Number of significant DEGcts. b-g: Volcano plots of DEGcts with the log2 fold change (logFC) on the x-axis and adjusted P value on the y-axis. *Please note that axis scales are not uniform*. (b) Non-pregnant vs E6.5 (n = 1167), c) E6.5 vs E8.5 (n = 546), d) E8.5 vs E10.5 (n = 659), e) E10.5 vs E12.5 (n = 189), f) E12.5 vs E15.5 (n = 203) and g) E15.5 vs E17.5 (n = 59). The significantly differentially expressed genes are depicted by blue dots. (Benjamini- Hochberg adjusted pvalue <0.05). The top ten most up-regulated and down-regulated genes (by log2 fold change) in each comparison are labelled by gene name. Volcano plots of DEGcts in high resolution are present in supplementary figures (S2–S7 Figs).

Identification of upstream regulators is based on the differential expression between consecutive time points (DEGcts) of progressing gestation. The number of significant DEGcts are illustrated in Fig 3A (S3 Table, pathway enrichment analysis for consecutive time point comparisons are present in S4 Table). The NP to E6.5 comparison yields the highest number of DEGcts. The volcano plots (Fig 3B–3G) visualize the spread of DEGcts, with higher lfc observed in earlier stages compared to the later time points. When comparing gene expression of the non-pregnant uterus to that at E6.5 *LOC100047285*, *Prss28*, *Spink3* and *Nppa* with a lfc change >5 are highly upregulated at E6.5 compared to NP, and are subsequently down regulated at E8.5 compared to E6.5 (Fig 3C).

**Fig 4:**
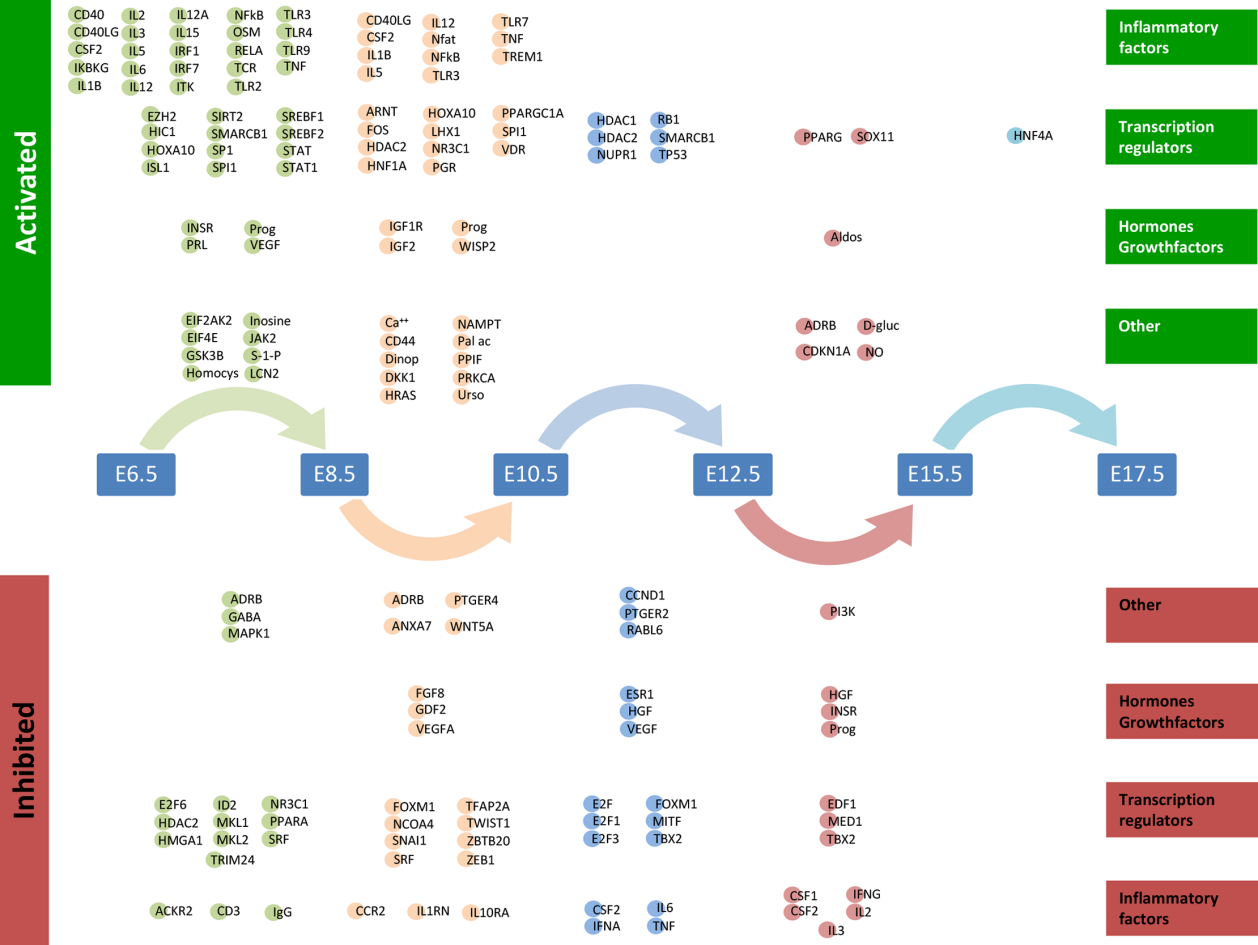
Predicted upstream regulators of differentially expressed genes between consecutive time points (DEGcts). Time window specific identification of upstream analysis of the DEGcts using Ingenuity Pathway Analysis (IPA). The coloured circles are upstream regulators identified for each comparison of consecutive time points with an activation or inhibition Z score of respectively > 2.0 or < (-2.0) (S5 Table). Circle colours correspond to colours of the arrows linking the consecutive time points. Active upstream regulators are displayed at the top of the **Figure** and the inhibited upstream regulators are at the bottom of the **Figure**. The upstream regulators are grouped into four different categories: ‘Inflammatory factors’, ‘Transcription regulators’, ‘Hormones and growth factors’ and ‘Other’. Aldos = Aldosterone, D-gluc = D-glucose, Dinop = Dinoprost, Homocys = Homocysteine, NO = Nitric oxide, Pal ac = Palmitic acid, Prog = Progesterone, S-1-P = Shingosine-1-phosphate, Urso = Ursodeoxycholic acid.

Ingenuity Pathway Analysis (IPA) [38] was applied to define upstream regulators relevant for each set of DEGcts (S5 Table). The upstream regulators identified for each DEGct comparison represent 3 main categories of functionality as illustrated in Fig 4. The top 5 upstream regulators for each comparison based on their Z-scores is illustrated in Table 1. From E15.5 to E17.7 the number of differentially expressed genes is at its nadir with only HNF4A predicted as an active upstream regulator.

**Fig 5:**
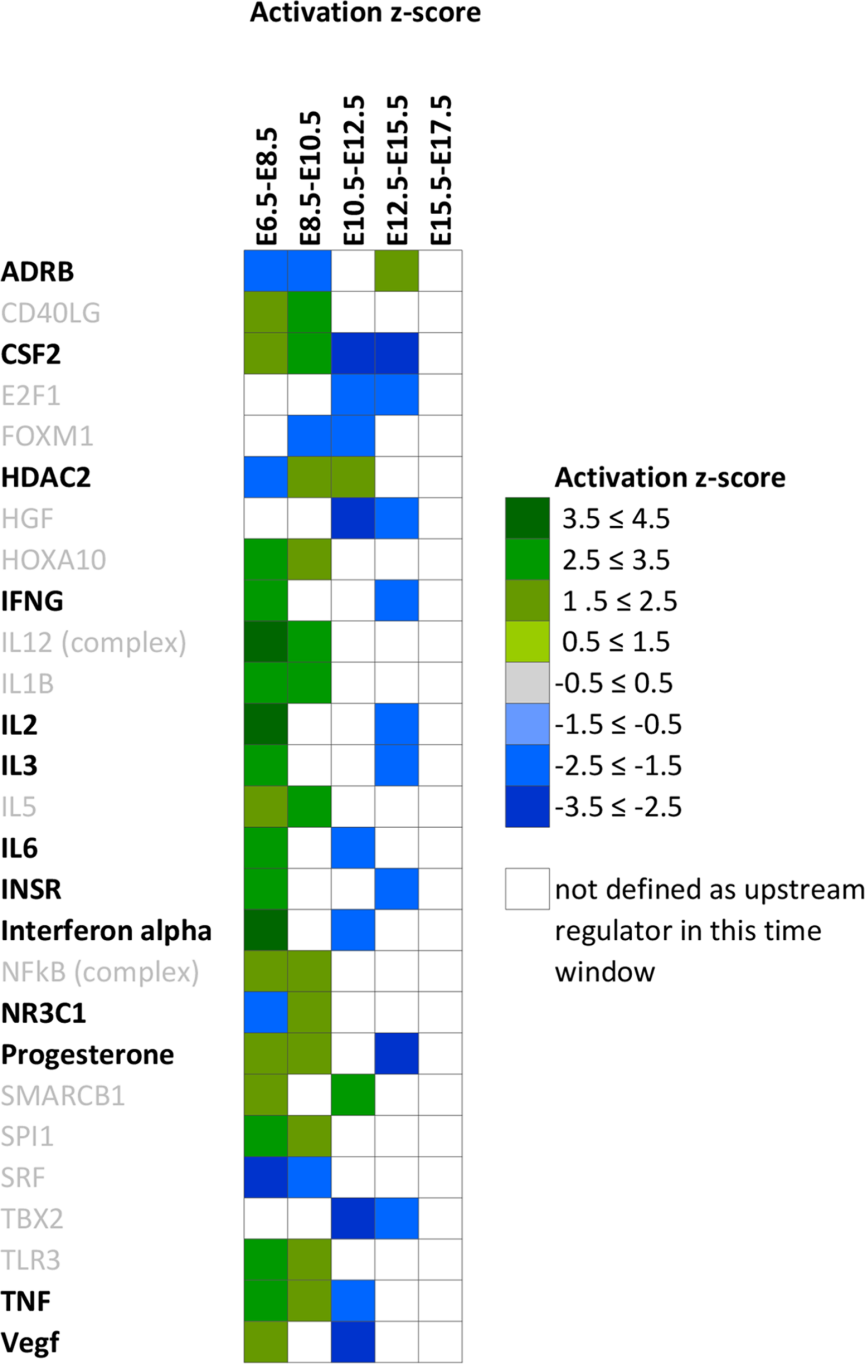
Predicted repetitive upstream activators. Depicted are upstream activators with an activation or inhibition Z score of respectively > 2.0 or < (-2.0) present in at least 2 time window comparisons. Positive Z-scores are depicted in green, negative Z-scores are depicted in blue.

**Table 1:** Activated and inhibited upstream regulators for each consecutive time point comparison.

Compared embryonal time points	Activated upstream regulators	Z-score	Inhibited upstream regulators	Z-score
E6.5-E8.5	IL2	4.156	TRIM24	-3.359
	IL12(complex)	3.663	PPARA	-2.618
	Interferon alpha	3.538	MKL1	-2.571
	TNF	3.430	SRF	-2.554
	SREBF1	3.347	ADRB	-2.490
E8.5-E10.5	IL5	3.347	PTGER4	-3.000
	CD40LG	2.757	IL10RA	-2.846
	IL1B	2.715	SNAI1	-2.577
	CSF2	2.709	TWIST1	-2.514
	PGR	2.688	GDF2	-2.456
E10.5-E12.5	NUPR1	3.771	TBX2	-3.450
	TP53	3.301	HGF	-3.267
	SMARCB1	2.917	PTGER2	-3.000
	RB1	2.613	CSF2	-2.819
	HDAC1	2.236	Vegf	-2.704
E12.5-E15.5	PPARG	2.506	CSF2	-2.926
	D-glucose	2.412	progesterone	-2.547
	ADRB	2.236	HGF	-2.402
	aldosterone	2.207	IFNG	-2.317
	CDKN1A	2.168	TBX2	-2.236
E15.5-E17.5	HNF4A	2.208		




Twenty-seven putative upstream regulators were predicted for more than one time window and 13 of them switch their direction of regulation during the gestational period investigated (Fig 5).

The important role of progesterone in the establishment of pregnancy and its functional withdrawal essential to the transition from uterine quiescence to overt synchronized contractions is well known. In the current dataset, we identify progesterone as one of the active hormonal upstream regulators both at E6.5 to E8.5 and E8.5-E10.5. Progesterone is not present as predicted upstream regulator from E10.5 to E12.5 and comes up again at E12.5-E15.5 with a negative Z score predictive value, showing the switch of this hormonal system to an inhibited state.

This finding draws attention to the E10.5-E12.5 window for which 22 upstream regulators are predicted, 6 of which the function is predicted as activated and 16 are predicted as inhibited. NUPR1, has the highest positive predictive Z score at E10.5 to E12.5. TBX2, with the highest negative predictive Z score at E10.5 to E12.5 is also listed as an inhibited upstream regulator for the E12.5 to 15.5 window of differential gene expression.

## Discussion

Parturition is a natural phenomenon at the end of mammalian pregnancy when fetal maturation is compatible with extra-uterine life. Currently, both preterm labour and functional labour dystocia are clinical challenging obstetrical problems for which only limited therapeutics are available. Functional progesterone withdrawal, prostaglandin synthesis, oxytocin receptor activity and the expression of connexin-43 are all essential to activate voltage gated calcium channels and induce the high connectivity between uterine myocytes required for functional labour. The main goal of the current study is to identify upstream regulators that play a role in the initiation of parturition and the timely regulation of all pathways essential to attain synchronized contractions.

In the current study we collected uterine tissues from E6.5 to E17.5 in pregnant FVB mouse as well as from control non-pregnant mice and determined gestational time-point specific gene expression profiles using micro-array. Time points after E17.5 were not investigated as they have been previously well studied [32, 33, 35] and do not contribute to the aim of the current paper.

PCA shows proper clustering of gene expression profiles for the different sample time points.

Although the data from the non-pregnant mice uterus are not directly relevant for the identification of putative upstream regulators of labour they do demonstrate the validity of our approach. *LOC100047285*, *Prss28*, *Spink3* and *Nppa* that are upregulated (lfc >5) at E6.5 compared to non-pregnant, are down regulated at E8.5 compared to E6.5 (**Fig** 3C) indicating a rather stage specific flip of gene expression in early pregnancy.

*Prss28* (also known as *Isp1* implantation serine protease1) and *Spink3* (Secretory peptidase inhibitor) have both been previously implicated in implantation related processes[43–45]. Also Proline rich acidic protein 1 *(Prap1)* that is upregulated at E6.5 compared to non-pregnant has well-established functions in implantation [46].

Implantation requires an adaptive immune response where the maternal immune system should accept the fetus as a partial allograft. As elucidated in the results section, progesterone and inflammatory mediators (NFKB, STAT1, TLR2, TLR4, TNF, IL2, IL3, and IL6) are active upstream regulators at E6.5-E8.5–10.5 (**Fig** 4 and Table 1). Coinciding with progesterone withdrawal, activation of the inflammatory response is a well-known mechanism in the activation of labour. In the current dataset the activating inflammatory mediators between E6.5 and E10.5 disappear at E10.5 after which a subset reappears as inhibited upstream activators.

The immune related role of CSF2 (Predictive Z-score -2.82) in early pregnancy has been well described [47]. In the current study, CSF2 is predicted as an active upstream regulator from E6.5 to E10.5 after which it comes up as inhibited, suggesting a possible role in prevention of inflammatory response in mid-pregnancy. At the same time, active upstream regulators at E12.5-E15.5 such as Nitric oxide (Z-score 2.00) [48, 49], beta-adrenergic receptor complex ADRB (Z-score 2.24) [50, 51], PPARG (Z-score 2.51) [52–54] are established mediators of uterine quiescence.

The gene expression clusters during gestation shed light on the complexity of gene networks and the molecular cascades required for the on-going pregnancy and in preparation for parturition. Particularly, clusters 3, 4, 5, 6 & 7 that show significant enrichment in specific biological processes. Cluster 3 enriched in processes involving cellular mechanisms of regulation of gene expression and protein synthesis decreases from early to late pregnancy.

Cluster 4 & Cluster 5 indicate a suppression of T-cell receptor signaling/T cell activation during mid-pregnancy. On the other hand cluster 6 which increases from early to late pregnancy shows an enrichment in Toll-like receptor signaling some of which have been known to play an active part in labour [55, 56].

Functional progesterone withdrawal and induction of prostaglandins is one of the known key regulators to the transition from quiescence to overt labour in both mice and humans [57–59]. Previous studies in mice have shown that the increased expression of the oxytocin receptor and connexin-43 after E15.5 are preceded by the downregulation of the zinc finger transcription factors *Zeb1* and *Zeb2* evoked by the upregulation of the miR-200 family that perform a central role in the process of progesterone mediated uterine quiescence [26, 35]. We observe *Zeb1* in cluster 1 which decreases from early to mid-pregnancy.

IPA defines progesterone as active upstream regulator in both the E6.5 to E8.5 and the E8.5 to E10.5 time windows fitting its well established role in the maintenance of pregnancy. Progesterone switches to an inhibited upstream regulator in the E12.5 to E15.5 window indicating functional progesterone withdrawal and highlighting the interest for the E10.5 to E12.5 time window.

From studies in C57Bl/6 mice it is evident that the transcription factor E47-like factor 3(*Elf3*) increases from E13.5 to E16.5 [33]. *Elf3* is able to regulate the promoter activity of *Ptgs1* (also known as *Cox-1*) that compared to E13.5 is upregulated at E15.5 and is responsible for the production of prostaglandins. Similarly, we observe *Elf3* in Cluster 6 and *Ptgs1* in Cluster 7 with a 1.7 lfc in expression during the E12.5 to E15.5 window in FVB mouse uterus. This again highlights the importance of the E10.5 to E12.5 time window for the identification of novel upstream regulators.

Previous studies on tumor protein p53 (*Trp53*), one of the active upstream regulators at E10.5 to E12.5 identified in the current study, have shown that mice with a conditional deletion of uterine *Trp53* have an increased incidence of preterm birth. In addition, 75% of dams with preterm birth also show signs of dystocia with prolonged labour over a period of 12 hours [30].

NUPR1 predicted as an active upstream regulator, (*Nupr1*) present in cluster 6 gradually increases from early to late pregnancy and TBX2 predicted as an inhibited upstream regulator (*Tbx2*) is present in cluster 1 decreases from early to late pregnancy. TP53 (*Trp53*) present in cluster 6 decreases towards late pregnancy. In addition to TP53, we also observe NUPR1, TBX2, HGF and PTGER2 as upstream regulators with an absolute predictive Z-score above 3 identified at E10.5 to E12.5. They are strong candidates to play an important role in regulating the molecular events that are necessary for the transition from uterine quiescence to initiation of parturition.

### Limitations of the study

The limitations of the study are the probe coverage and the fact that gene expression is unable to quantify regulation at the translational or posttranslational level. Given the homogeneity of gene expression within samples from a particular gestational time point and the profound positive correlations with previous experimental observations this study is a valuable asset of genes playing a role in gestation and parturition.

## Supporting information

S1 Table Seven clusters. Each worksheet contains the mouse genes expressed in that specific cluster with scaled normalized relative gene expression data.
(XLS)

S2 Table Go enrichment of clusters. Each worksheet displays GO terms (Column A) of all biological processes per gene expression cluster. Adjusted P values <0,05 are considered statistically significant and are highlighted.
(XLS)

S3 Table Differentially expressed genes between consecutive time points (DEGcts). Each worksheet contains the DEGcts for the indicated specific time point comparison. Lfc expression level and adjusted P value are indicated. Lists are ranked by adjusted P-value. Data are used to construct Fig 3.
(XLS)

S4 Table Pathway enrichment for differentially expressed gene sets between non-pregnant uterus and different embryonic stages. Each worksheet contains enriched pathways specific the indicated time point comparison.
(XLSX)

S5 Table Defined upstream regulators. Upstream regulators corresponding to the differentially expressed genes defined for each consecutive time point comparison are listed. For each upstream regulator, the functional role and functional direction (either activating or inhibiting their downstream targets) as well as their downstream reported (human) genes are indicated.
(XLSX)

S1 Fig Definition of the optimal cluster number. Weighted gap statistic measures within-clusters homogeneity and determining the minimum of clusters (x-axis) that explains the highest fraction of variance between differential gene expression patterns (y-axis). The optimal number of clusters was defined based on the saturation point of the curve.
(PDF)

S2 Fig Volcano plots of DEGcts of non-pregnant versus E6.5 with the log2 fold change (logFC) on the x-axis and adjusted P value on the y-axis. Please note that axis scales are not uniform.
(TIF)

S3 Fig Volcano plots of DEGcts of E6.5 versus E8.5 with the log2 fold change (logFC) on the x-axis and adjusted P value on the y-axis. Please note that axis scales are not uniform.
(TIF)

S4 Fig Volcano plots of DEGcts of E8.5 versus E10.5 with the log2 fold change (logFC) on the x-axis and adjusted P value on the y-axis. Please note that axis scales are not uniform.
(TIF)

S5 Fig Volcano plots of DEGcts of E10.5 versus E12.5 with the log2 fold change (logFC) on the x-axis and adjusted P value on the y-axis. Please note that axis scales are not uniform.
(TIF)

S6 Fig Volcano plots of DEGcts of E12.5 versus E15.5 with the log2 fold change (logFC) on the x-axis and adjusted P value on the y-axis. Please note that axis scales are not uniform.
(TIF)

S7 Fig Volcano plots of DEGcts of E15.5 versus E17.5 with the log2 fold change (logFC) on the x-axis and adjusted P value on the y-axis. Please note that axis scales are not uniform.
(TIF)
