# Supplementary figures and images for: In silico analysis of the Mus musculus uterine gene expression landscape during pregnancy identifies putative upstream regulators for labour
Source: PLoS One. 2018 Sep 20;13(9):e0204236. doi: 10.1371/journal.pone.0204236 (PMC6147639; doi:10.1371/journal.pone.0204236)

Optimal number of clusters

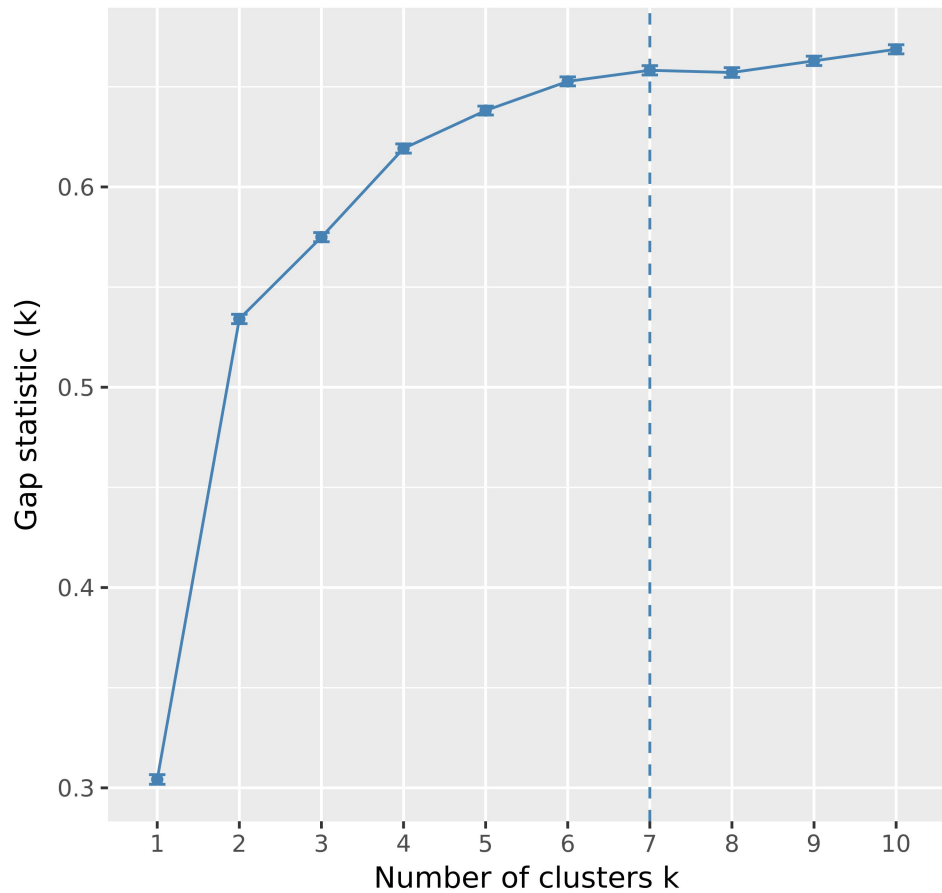

Supplement: S1 Fig — Weighted gap statistic measures within-clusters homogeneity and determining the minimum of clusters (x-axis) that explains the highest fraction of variance between differential gene expression patterns (y-axis). The optimal number of clusters was defined based on the saturation point of the curve. (PDF) [file pone.0204236.s006.pdf]

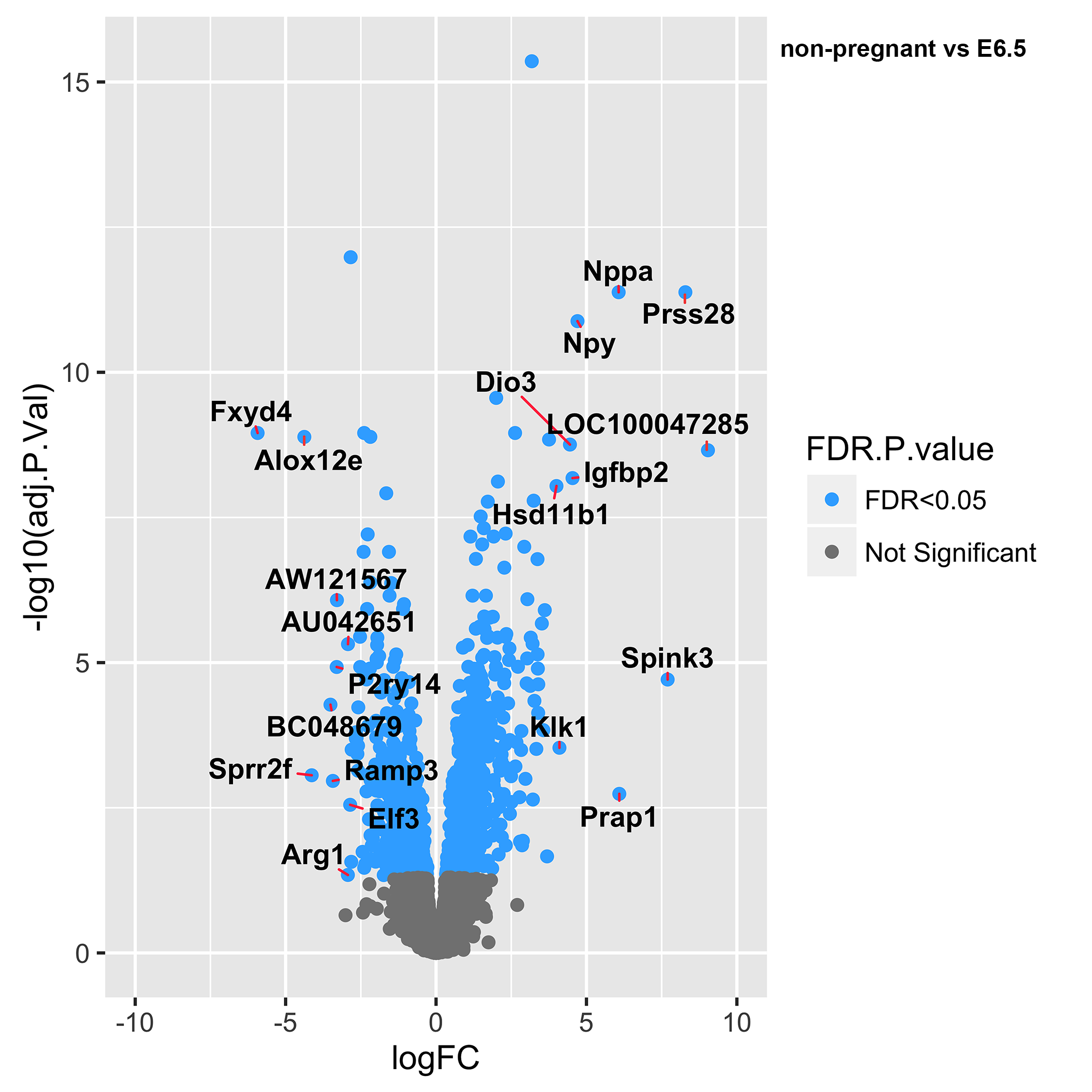

Supplement: S2 Fig — Please note that axis scales are not uniform. (TIF) [file pone.0204236.s007.tif]

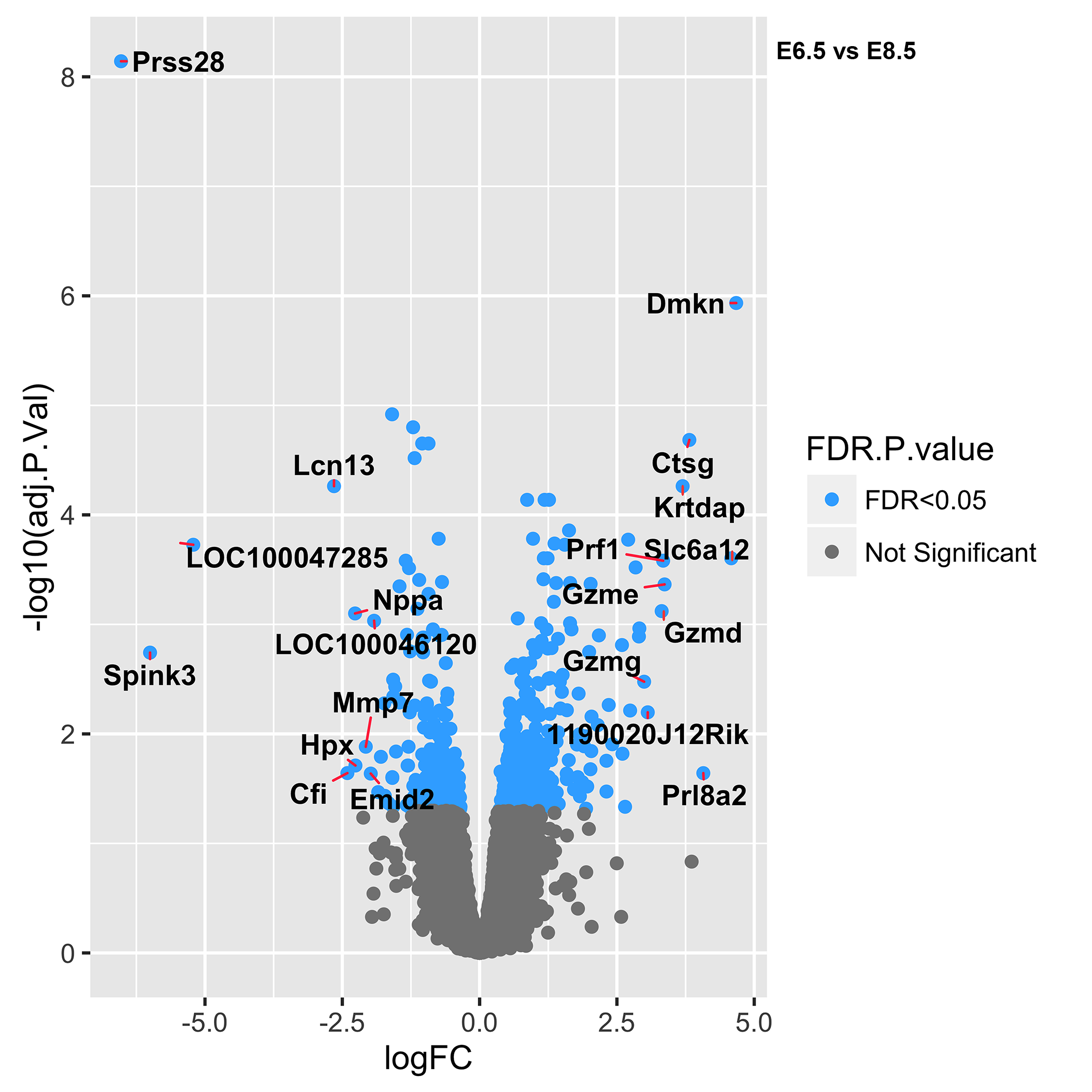

Supplement: S3 Fig — Please note that axis scales are not uniform. (TIF) [file pone.0204236.s008.tif]

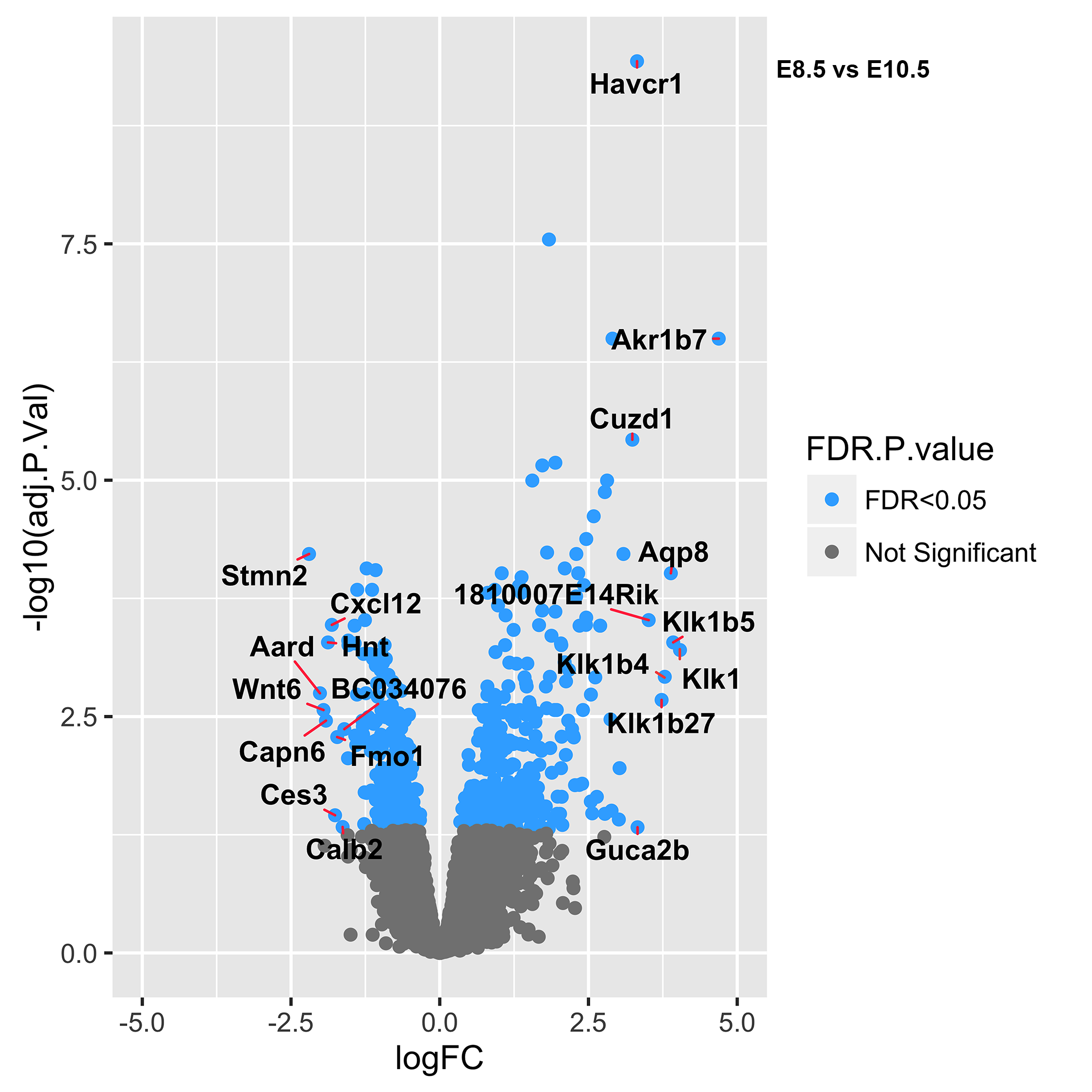

Supplement: S4 Fig — Please note that axis scales are not uniform. (TIF) [file pone.0204236.s009.tif]

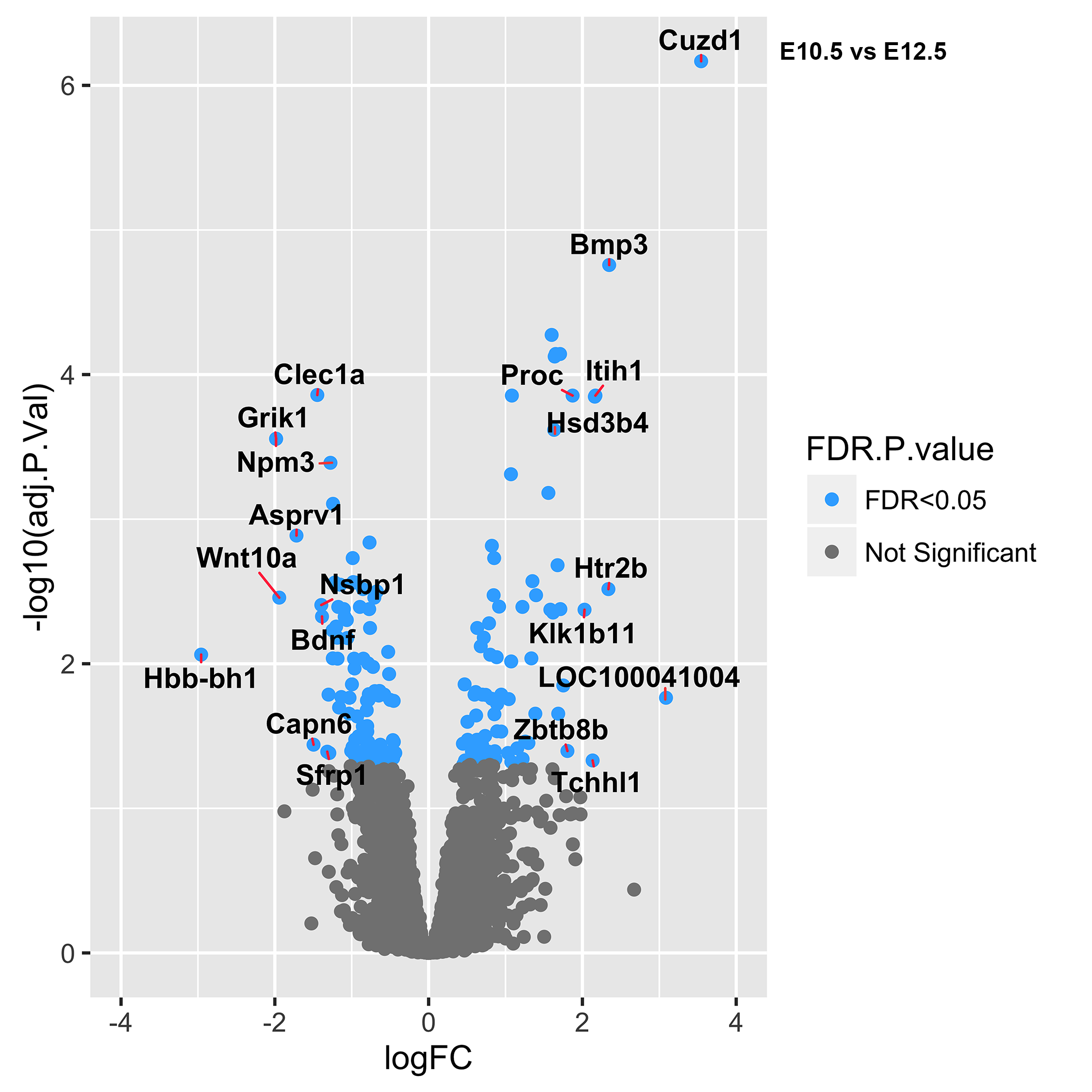

Supplement: S5 Fig — Please note that axis scales are not uniform. (TIF) [file pone.0204236.s010.tif]

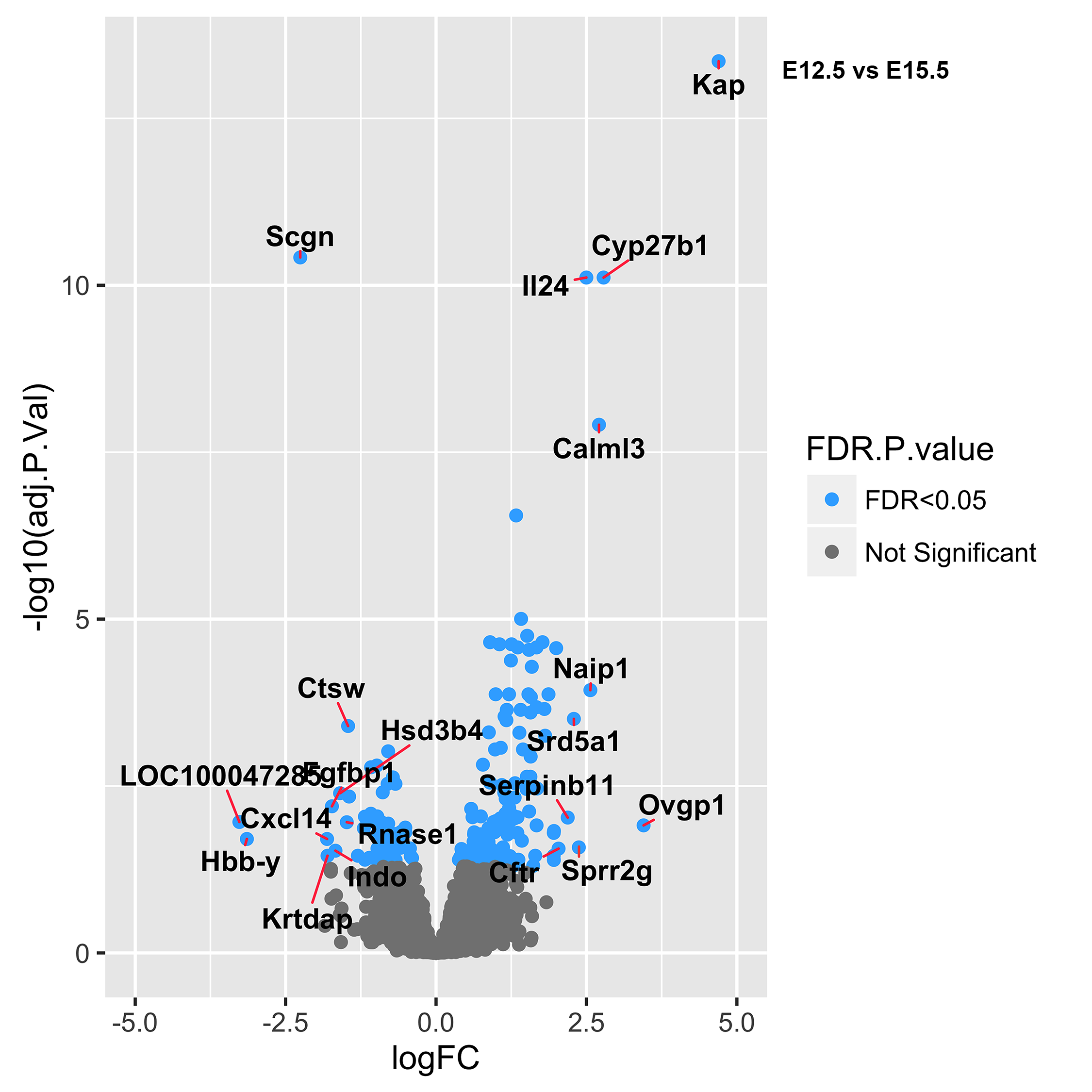

Supplement: S6 Fig — Please note that axis scales are not uniform. (TIF) [file pone.0204236.s011.tif]

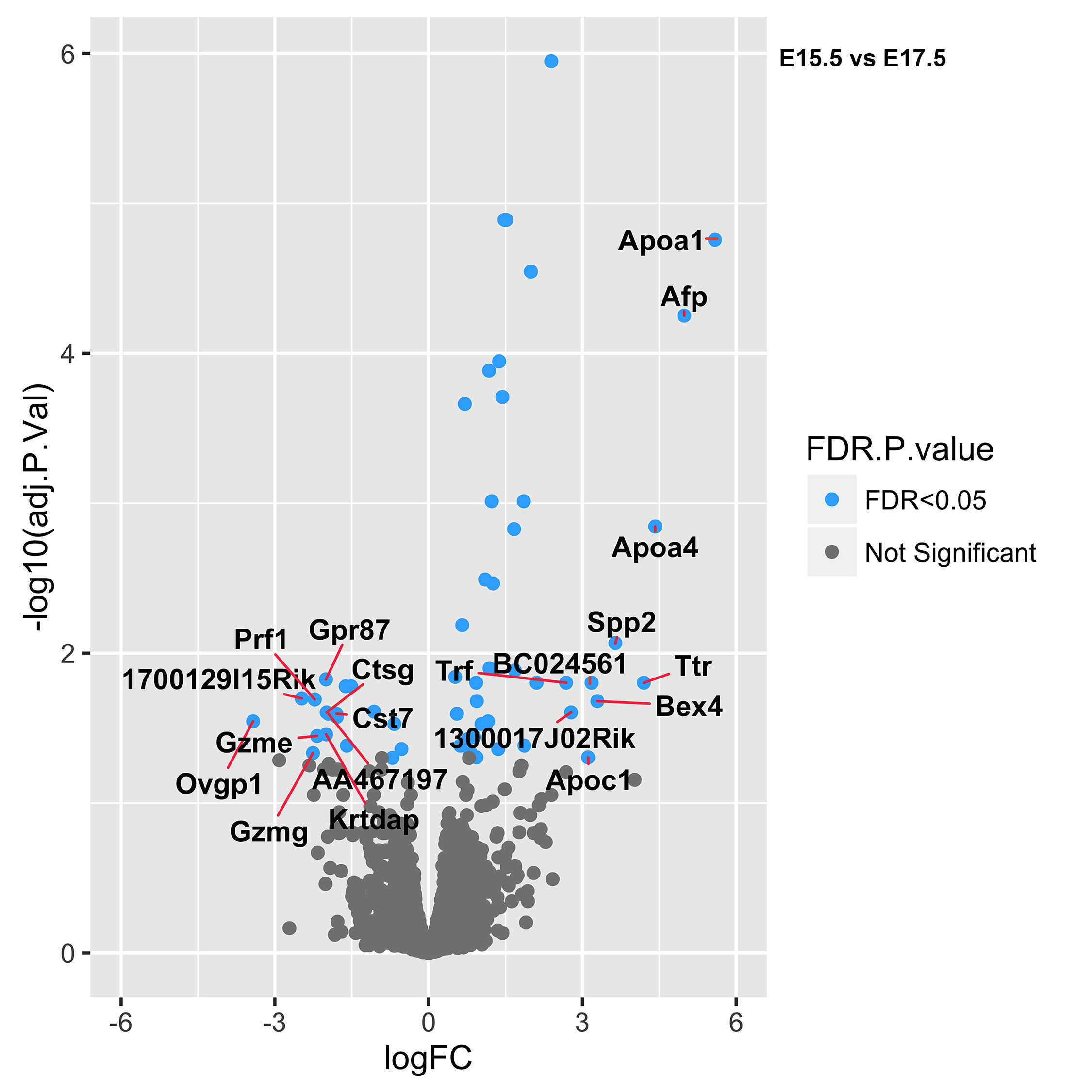

Supplement: S7 Fig — Please note that axis scales are not uniform. (TIF) [file pone.0204236.s012.tif]
